# Temporal Gene Expression and DNA Methylation during Embryonic Stem Cell Derivation

**DOI:** 10.22074/cellj.2018.5482

**Published:** 2018-05-28

**Authors:** Azam Samadian, Mahdi Hesaraki, Sepideh Mollamohammadi, Behrouz Asgari, Mehdi Totonchi, Hossein Baharvand

**Affiliations:** 1 Department of Stem Cells and Developmental Biology, Cell Science Research Center, Royan Institute for Stem Cell Biology and Technology, ACECR, Tehran, Iran; 2 Department of Genetics, Reproductive Biomedicine Research Center, Royan Institute for Reproductive Biomedicine, ACECR, Tehran, Iran; 3 Department of Developmental Biology, University of Science and Culture, Tehran, Iran

**Keywords:** DNA Methylation, MEK Inhibitor, Mouse Embryonic Stem Cells, R2i, TGFβ Inhibitor

## Abstract

**Objective:**

Dual inhibition of mitogen-activated protein kinase (MAPK) kinase (also known as MEK) and transforming growth factor β (TGFβ) type I receptors by PD0325901 and SB431542, known as R2i has been introduced as a highly efficient approach to the generation of mouse embryonic stem cells (ESC). In the present study, we investigated the molecular mechanisms underlying ESC derivation in the R2i condition.

**Materials and Methods:**

In this experimental study, zona-free whole E3.5 blastocysts were seeded on mouse embryonic fibroblast (MEF) feeder cells in both R2i and serum conventional media. The isolated inner cell mass (ICM), ESCs and the ICM-outgrowths were collected on days 3, 5 and 7 post-blastocyst culture for quantitative real time- polymerase chain reaction (qRT-PCR) analysis as well as to assess the DNA methylation status at the time points during the transition from ICM to ESC.

**Results:**

qRT-PCR revealed a significantly higher expression of the pluripotency-related genes (*Oct4, Nanog, Sox2, Rex1, Dppa3, Tcf3, Utf1, Nodal, Dax1, Sall4* and *β-Catenin*) and lower expression of early differentiation genes (*Gata6, Lefty2* and *Cdx2*) in R2i condition compared to the serum condition. Moreover, the upstream region of Oct4 and Nanog showed a progressive increase in methylation levels in the upstream regions of the genes following in R2i or serum conditions, followed by a decrease of DNA methylation in ESCs obtained under R2i. However, the methylation level of ICM outgrowths in the serum condition was much higher than R2i, at levels that could have a repressive effect and therefore explain the absence of expression of these two genes in the serum condition.

**Conclusion:**

Our investigation revealed that generation of ESCs in the ground-state of pluripotency could be achieved by inhibiting the MEK and TGF-β signaling pathways in the first 5 days of ESC derivation.

## Introduction

Mouse embryonic stem cells (ESCs) are pluripotent cells that were initially isolated from blastocysts and cultured on cell cycle arrested mouse embryonic fibroblast (MEF) feeder cells using fetal calf serum (FCS) (1). Then, MEF and FCS were later replaced with leukemia inhibitory factor (LIF) (2) and bone morphogenetic protein 4 (BMP4) respectively (3). Later, the ground-state hypothesis of pluripotency by Smith and colleagues suggested that the chemical inhibition of endogenous differentiation signals, fibroblast growth factor 4 (FGF4) using PD0325901 and glycogen synthase kinase 3 (GSK3), with CHIR99021, known as 2i, can maintain cultured ESCs in the pluripotent state (4). 

In another approach to the preserve ground state, Hassani et al. (5, 6), reported that dual inhibition of mitogenactivated protein kinase (MAPK) kinase (also known as MEK) and transforming growth factor ß (TGFß) type I receptors with PD0325901 and SB431542, known as R2i, results in highly-efficient generation of mouse ESCs even from refractory strains and single blastomeres (7). This medium also supports the efficient establishment of embryonic germ cell (EGC) lines from the primordial germ cells of mice (8) and rats (9). 

In contrast to when the multifunctional GSK3 protein is inhibited in 2i, under R2i, the ESCs show better homogeneity (i.e. cell-to-cell conformity in expression of pluripotency genes such as Nanog and Dppa3), genomic integrity, and ground-state pluripotency. In which a less complex condition is required for investigating the molecular mechanisms of ground-state pluripotency (5, 6, 10).

These advantages of R2i prompted us to further assess the molecular mechanisms that underlie the transition from inner cell mass (ICM) to ESC. Recently, Totonchi et al. (11), reported the key genes involved in the transition from ICM to ESC via temporal microarray gene expression analysis. They also used deep hairpin bisulfite sequencing (DHBS) to show the methylation of individual CpG sites for three classes of repetitive elements, micro satellites (mSats), the 5´ untranslated region of L1Md_Tf (L1), and a class of LTR-retrotransposons (IAP-LTR1) (11, 12). Their results indicated that DNA methyltransferases play a pivotal role in efficient ESC generation. However, the exact molecular mechanisms through which the derivation of ESCs takes place still needed to be clarified. 

Here, we assessed the expression of key genes involved in pluripotency, epigenetic and early differentiation using quantitative real time-polymerase chain reaction (qRT-PCR) Then, the DNA methylation status of cytosine guanine dinucleotides (CpG) upstream of the transcription starting site of two pluripotency-related genes (*Oct4* and *Nanog*) was determined using bisulfite genomic sequencing. Data was collected at different time points during the transition from ICM to ESC in R2i and compared to results from the serum culture condition.

## Materials and Methods

### Mice, embryos and media 

We collected E3.5 blastocysts by flushing the uteri of BALB/c (for qRT-PCR analysis) and NMRI (for R2i time point analysis) mouse strains after superovulation. Immunosurgery was performed to isolate ICMs from the blastocysts. Derivation of ESCs was done by plating the zona-free whole E3.5 blastocysts on NMRI strain-derived MEF feeder cells in R2i and serum conventional medium, as previously described (13). R2i medium was composed of DMEM/F12 (Invitrogen, USA) and neurobasal (Invitrogen, USA) in a 1:1 ratio, 1% N2 supplement (Invitrogen, USA), 1% B27 supplement (Invitrogen, USA), 1% nonessential amino acids (Invitrogen, USA), 2 mM L-glutamine (Invitrogen, USA), 100 U/ml penicillin and 100 mg/ml streptomycin (Invitrogen, USA), 0.1 mM ß-mercaptoethanol (Sigma-Aldrich, USA), 5 mg/mL bovine serum albumin (Sigma-Aldrich, USA), 1000 U/mlLIF (Royan BioTech, Iran), 1 µM PD0325901 (Stemgent, USA) and 10 µM SB431542 (Sigma-Aldrich, USA). Serum medium consisted of knockout Dulbecco’s modified Eagle’s medium (Invitrogen), 15% fetal bovine serum (FBS, HyClone), 1% nonessential amino acids, 2 mM L-glutamine, 100 U/ml penicillin, 100 mg/ml streptomycin (Invitrogen, USA), 0.1 mM ß-mercaptoethanol, and 1000 U/ml mouse LIF. 

The isolated ICMs, ESCs and the ICM-outgrowths were collected on days 3, 5 and 7 post-blastocyst culture in three independent replicates and stored at -80oC. Each experimental group included 20 to 30 embryos/ outgrowths. All experiments were approved by the Ethical Committee of Royan Institute. 

### RNA isolation and quantitative real time-PCR

Total RNA was extracted from the three independent replicates using RNeasy micro kit (Qiagen, USA). The purity and concentration of the RNA was assessed and quantified by measuring the absorbance A260 nm/ A280 nm using a Biowave II spectrophotometer (WPA, Biochrom, UK). The quality and integrity of the total RNA was verified by electrophoresis. A total amount of 2 µg of total RNA was converted into cDNA using RevertAid cDNA synthesis kit and Random hexamer primers (Thermo Fisher Scientific, USA). 

Quantitative real time PCR was carried out using SYBR Green master mix (ABI, Step one plus, USA). Primers (Table S1) (See Supplementary Online Information at www.celljournal.org) were designed by Perlprimer software and checked in Gene Runner software (http:// www.generunner.com). The reactions were carried out in triplicates and qRT-PCR amplification was performed using the following program; stage1: 95oC for 10 minutes, stage 2 (40 cycles): 95oC for 10 seconds, 60oC for 60 seconds. The results were normalized against the reference gene (*Gapdh*) and compared with ICM. The relative quantification of gene expression was calculated using the ΔΔCt method. 

### DNA methylation assay

Pure DNA (1 µg) was treated with EpiTect Bisulfite Kit (Qiagen, USA). Semi-nested methylation specific primers (MSP) were designed for 2 promoter regions of two pluripotency-related genes (*Nanog* and *Oct4*), using Methprimer software. The primers used were:

Oct4 F1: 5´-GTTGTTTTGTTTTGGTTTTGGATAT-3´
  F2: 5´-ATGGGTTGAAATATTGGGTTTATTTA-3´
  R: 5´-CCACCCTCTAACCTTAACCTCTAAC-3´
Nanog F1: 5´-GAGGATGTTTTTTAAGTTTTTTTT-3´
  F2: 5´-AATGTTTATGGTGGATTTTGTAGGT-3´
  R: 5´-CCACCCTCTAACCTTAACCTC TAAC-3´

The PCR cycling program started at 95oC for 5 minutes, then 32 cycles of 95oC for 35 seconds, 53-54oC for 40 seconds, and 72oC for 35 seconds, followed by 72oC for 10 minutes. Subsequently, 1 µl of bisulfite-treated DNA from each sample was amplified by AmpliTaq Gold kit (Life technology, USA). The PCR products were cloned using a TA-cloning kit (Invitrogen, USA). Next, 15 single white colonies were selected and the cloned fragments were amplified with M13 universal primers. The PCR product of each selected clone was analyzed by BiQ Analyzer software. 

### Statistical analysis

The data were analyzed by one-way analysis of variance (ANOVA) test, followed by a Tukey post-hoc test for determination of significant differences among groups and are presented as mean ± SD. Differences among groups were considered statistically significant at P<0.05. 

## Results

### Temporal expression of pluripotency and differentiation-specific genes during transition from inner cell mass to embryonic stem cells 

**Fig.1:**
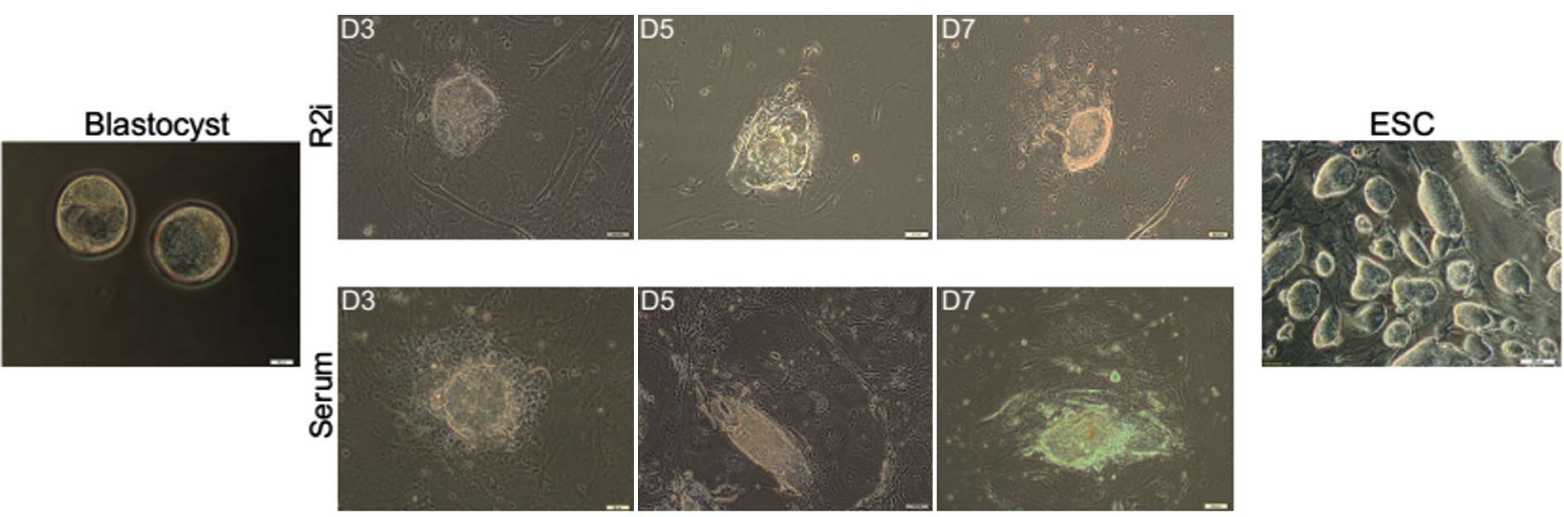
Morphology of embryonic stem cells (ESCs) during derivation under serum and R2i condition. Zona-free blastocysts isolated on embryonic day 3.5 were cultured on mouse embryonic fibroblast (MEF) feeders in serum and R2i. The inner cell mass (ICM)-outgrowth in R2i had a low density of trophectoderm cells and colonies were typically more compact as compared to those in serum.

**Fig.2:**
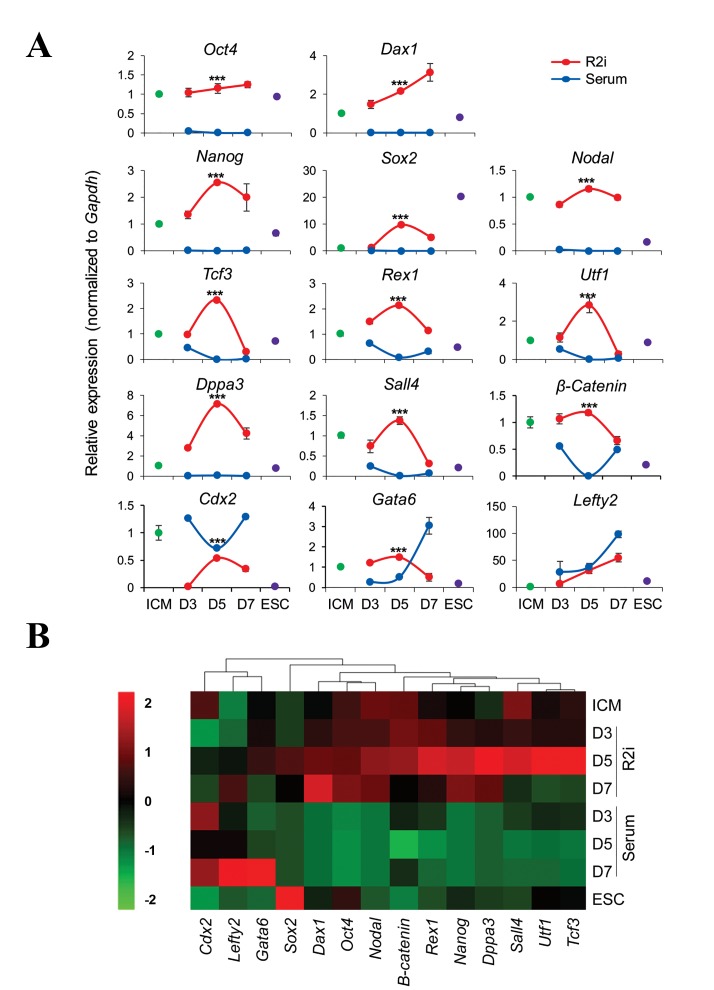
Temporal expression of pluripotency and differentiation-specific genes during embryonic stem cells (ESC) derivation. A. Gene expression analysis of inner cell mass (ICM)-outgrowths during ESC line derivation in serum and R2i. Quantitative real time-polymerase chain reaction (qRT-PCR) of related genes was performed for ICM-outgrowths on days 3, 5 and 7 in the serum and R2i and ESCs derived in R2i condition (p4). There were three biological replicates. All biological replicates for the indicated time points were mixed and then the reactions were carried out in technical triplicates (***; P<0.001) and B. Heat map showing clustering and variations in gene expression at indicated time points. It reveals that the expression levels of most pluripotency-related genes on day 5 are higher than those of days 3 and 7 in R2i.

Whole zona pellucida-free blastocysts were plated ontomitotically inactivated MEF feeder layer in R2i and serumconditions. The blastocyst-outgrowths in R2i culture have atypical compact morphology as opposed to those cultured in theserum medium. Also, it seems the number of trophectodermlike cells around attached blastocyst outgrowths decreased inR2i compared to serum (Fig .1). Next, to assess the temporalexpression of key pluripotency-related genes during theprocedure of mouse ESC establishment, we gently isolatedthe ICM outgrowths with a Pasture pipette on days 3, 5, and 7 after seeding the blastocysts. Then, qRT-PCR wasperformedto measure the expression of pluripotency markersnamely, *Oct4, Nanog, Sox2, Rex1, Dppa3, Tcf3, Utf1, Nodal, Dax1, Sall4* and *ß-Catenin* as well as early differentiation markers, *Gata6* as a primitive endoderm marker, *Lefty2* as a primitive mesoderm marker and *Cdx2* as a trophectoderm lineage marker (Fig .2). 

R2i caused a significantly higher expression of pluripotencyrelated genes during ESC derivation, while in serum, theexpression of these genes in outgrowths was not detected orwas at very low levels. We observed two distinct expressionpatterns for the genes in R2i codition. In the first group, theexpression continuously increased during derivation (*Oct4* and *Dax1*), while *Nanog, Sox2, Nodal, Dppa3 Tcf3, Rex1, Utf1,* and *ß-Catenin* were upregulated until day 5 and downregulatedafterward. In addition, the early lineage differentiation genes were expressed at lower levels under the R2i condition compared to serum (P<0.001, Fig .2A). 

Hierarchical clustering and heatmap analysis showedthat the expression of most pluripotency-related genes wasincreased in R2i compared to the ICM and the highest level of gene expression was observed on day 5 (Fig .2B).

### DNA methylation status of Oct4 and Nanog promoters and the expression of epigenetic-associated genes during embryonic stem cells derivation 

**Fig.3:**
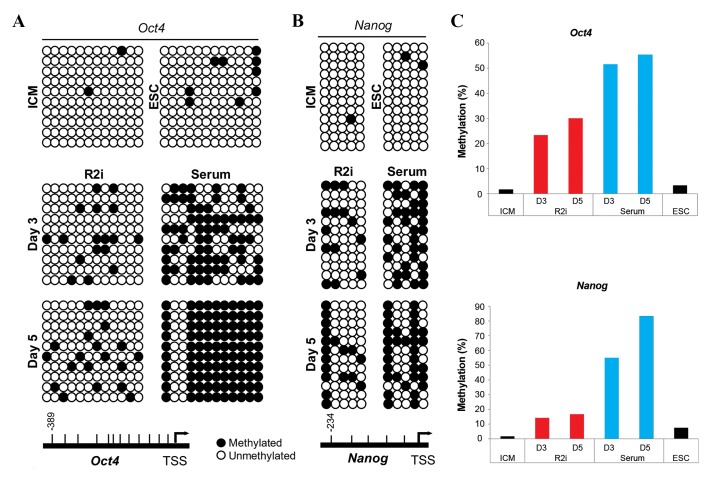
DNA methylation status of Oct4 and Nanog promoter s during embryonic stem cell (ESC) derivation. We analyzed the twelfth and tenth CpGs which are located in the promoter regions of A. Oct4, B. of each sample using bisulfite sequencing. DNA methylation profile on days 3 and day 5 were determined under both serum and R2i conditions. Under R2i condition, samples were hypomethylated compared to serum. Closed circles represent methylated CpGs, and open circles represent unmethylated CpGs, and C. Comparison of DNA methylation under the two conditions during transition from inner cell mass (ICM) to ESC.

Bisulfite sequencing was used to evaluate the methylation status of the twelfth and tenth CpGs in the promoter regions of the pluripotency-associated genes, *Oct4* and *Nanog* respectively. Based on our data, the promoters of these genes were highly unmethylated during the transition from ICM to ESC in R2i condition whereas CpG dinucleotides of the regions in outgrowths were highly methylated in serum condition (Fig .3). These findings indicate that these promoters might be more active under R2i.

**Fig.4:**
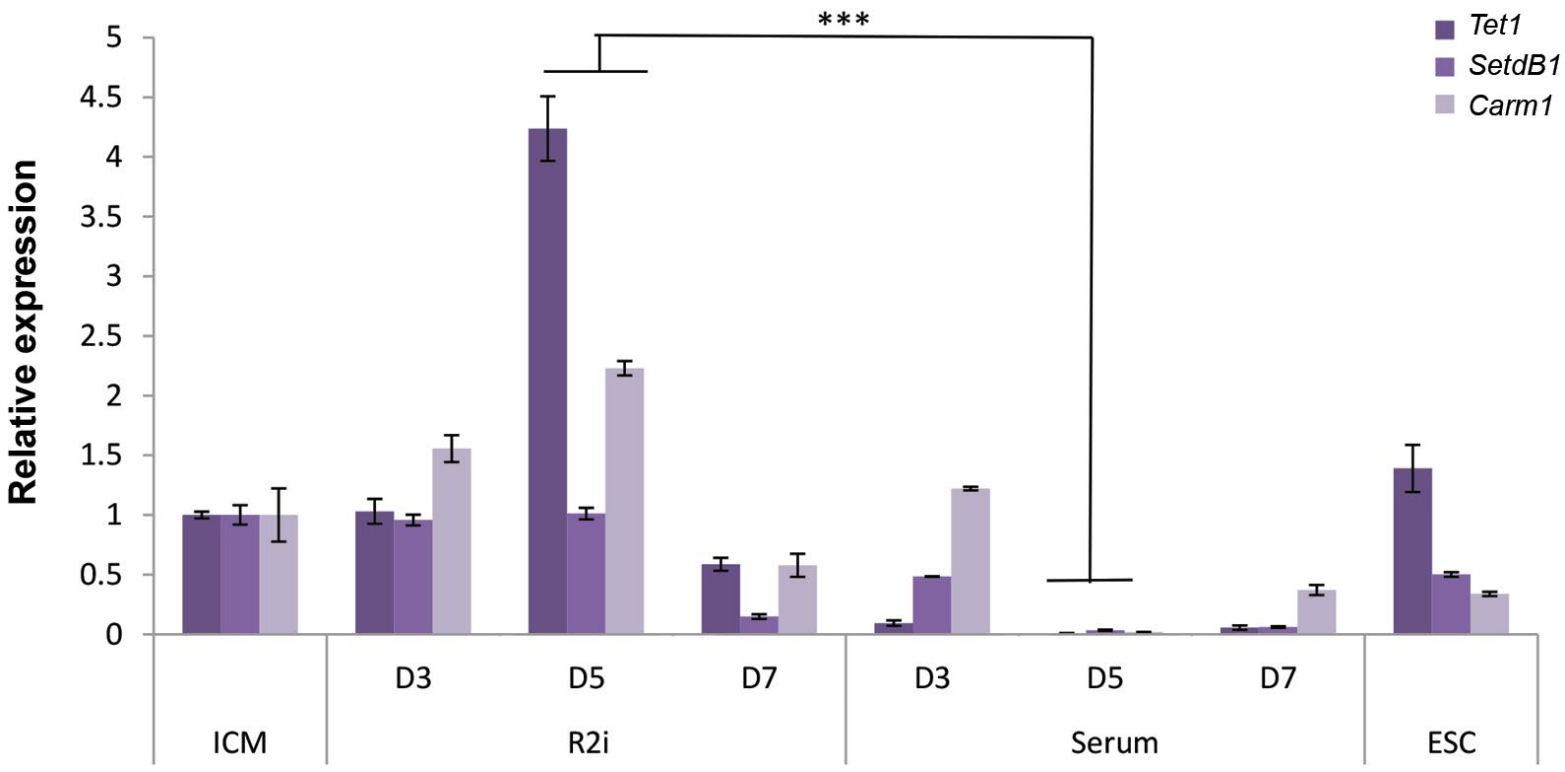
The expression of epigenetic-associated genes during embryonic stem cells (ESC) derivation. R2i maintains the expression of epigenetic-relatedgenes such as Tet1, Carm1 and SetdB1 in inner cell mass (ICM)-outgrowths. The maximum level of gene expression was observed on day 5 of thederivation process. There were three biological replicates. All three biological replicates were mixed at indicated time points and then the reactions werecarried out in technical triplicates (***; P<0.001 as compared to ICM).

On the other hand, relative expression of epigenetic-related genes (*Tet1, Carm1* and *Setdb1*) showed a significant up- regulation under R2i (P<0.001, Fig .4). Notably, the expression of these genes was upregulated in a similar manner to that of the pluripotency-related genes in day 5.

### Efficient embryonic stem cells generation after 5 days of treatment with R2i 

**Fig.5:**
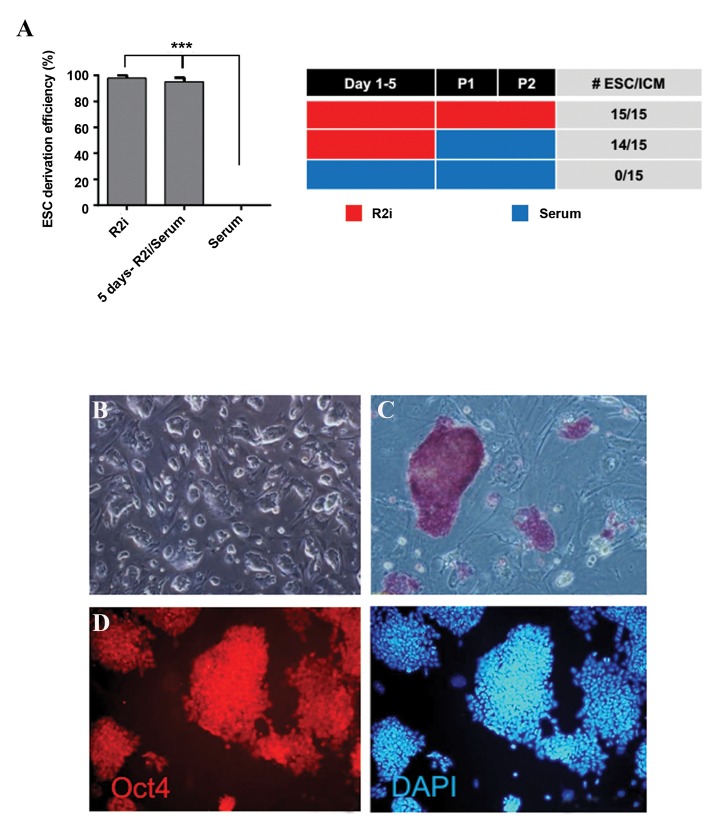
Efficiency of embryonic stem cells (ESC) derivation upon 5 days R2i treatment. A. Left and right, blastocysts were treated for 5 days in R2i and thencultured in serum. Zona-free blastocysts were cultured on feeder cells in the presence of R2i, 5 days in R2i and then cultured in serum (5 days-R2i/serum) and entirely in serum condition. On day 7, the individual outgrowths were trypsinized and replated on fresh mouse embryonic fibroblast (MEF) in 24-wellplate. Following 5 days, packed dome ESC-like colonies could be identified. ESC generation efficiency analysis showed that 5 days in the R2i condition issufficient to establish cell lines compare to serum. One-way ANOVA with Tukey post-hoc test was performed (***; P<0.001), B. Phase contrast of the new ESC line (scale bar; 200 µm), C. Alkaline Phosphatase (ALP) staining (scale bar; 100 µm), and D. Immunofluorescence labeling for Oct4 counterstained for DAPI (scale bar; 100 µm).

As early evidence of high expression levels of pluripotency-association genes and hypomethylated DNA was found in R2i, we sought to determine whether ESCs could be established if we cultured ICM in R2i for 5 days and then continued the remainder of the culture in serum (5 days-R2i/serum). Concordantly, 3 groups of zona-free blastocysts (NMRI strain) were cultured on feeder cells (15 embryos for each group); entirely in R2i culture medium, 5 days in R2i and then serum and also solely in the serum condition. On day 7, the individual ICM outgrowths were picked from the outgrown trophectoderm using a Pasteur pipette and subsequently trypsinized (trypsin/EDTA, 0.05% w/v) and replated on freshly seeded MEF in 24-well plates. After 5 days, typical packed domed ESC-like colonies could be identified. The efficiency in the generation of ESCs in R2i was 100% and ~ 94% for 5 days-R2i/serum while in the serum condition, ESC colonies did not appear (Fig .5A). The ESCs passaged easily and showed dome-shaped colony morphologies (Fig .5B), high nuclear/cytoplasmic ratios, the ability to propagate following trypsin digestion and clonal growth from single cells while also displaying high levels of alkaline phosphatase activity (Fig .5C) and Oct4 expression. Therefore, 5 days treatment of whole blastocysts with R2i on MEF is sufficient for efficient generation of ESCs. 

## Discussion

In this study, we reported the gene expression and DNA methylation of ICM during ESC generation under dual inhibition of MEK and TGFß signaling pathways with PD0325901 and SB431542 (known as R2i) that resulted in efficient generation of the ground-state pluripotency. R2i provides high genomic stability and an efficient transition from ICM to ESC (6). These advantages enable us to study the molecular mechanisms during ESC derivation. We analyzed the morphology of ICM-outgrowths on the MEF feeder layer in both, serum and R2i media. ICM-Outgrowths in serum had a larger fraction of trophoectoderm cells, while under R2i, the proliferation of these cells appeared to be inhibited. Therefore, we could conclude that ESC derivation on feeder cells in R2i medium reduces proliferation of trophoectoderm cells. In addition, the ICM-outgrowths were more compact and homogenous in comparison with the serum/LIF condition. 

Next, we designed experiments to develop an appropriate strategy to explore genetic and epigenetic mechanisms that underlie ESC derivation. Here, we found that R2i significantly promotes upregulation of the pluripotency-related genes (*Oct4, Nanog, Sox2, Rex1, Dppa3, Tcf3, Utf1, Nodal, Dax1, Sall4* and *ß-Catenin*) and downregulates early differentiation genes (*Gata6, Lefty2* and *Cdx2*). Previous studies have reported that increased expression of *Dax1* in ESC, led to an increased expression of *Oct4* (14, 15). Likewise, Oct4 can bind to the promoter region of Dax1 and regulate its expression level (16). It has been shown that a balanced expression of *Oct4*, probably plays an important role in maintaining pluripotency (17). In addition, it was indicated that *Gata6* and *Cdx2* were downregulated during ICM outgrowth (18). Therefore, under the R2i condition, the ground-state of pluripotency during transition from ICM to ESC was maintained through the suppression of differentiation- related pathways and enhancement of the expression of pluripotency-affiliated genes in ESCs (5-11, 19).

Moreover, we found that the promoter regions of pluripotent-associated genes, Oct4 and Nanog, of ICM-outgrowths were significantly hypomethylated under R2i compared to the serum condition during the early days of ESC derivation. Moreover, we found that the genome of ESCs was hypermethylated in selected regions compared to ICM cells. Our data showed that DNA methylation status in ESCs is similar in relation to in line with the indings of a comparison between 2i and R2i (20, 21). These patterns of DNA methylation have an essential role in the establishment of pluripotency under R2i (22, 23). We found that inhibition of DNA methylation by a methyltransferase inhibitor, RG-108 resulted in efficient generation of ESCs under R2i condition (11). 

The expression of epigenetic-related genes *Tet1, Carm1* and *Setdb1* was also significantly upregulatedunder the R2i condition. DNA methylation and the expression of epigenetic modifiers have beendemonstrated in early embryo development and long-term maintenance of pluripotent cells (12, 19,24-27). Previously, we had demonstrated that the expression of epigenetic-associated genes such as *Dnmt3b, Dnmt3l, Chd8, Mtss1, Suz12, Eed, Wdr3,* and *Mat2b* was significantly enhanced in the intermediatestages of ESC establishment (11). Finally, we clearlydemonstrated that 5 days in the present of R2i withsupporting MEF (5 days-R2i) is sufficient for efficient establishment of ESCs.

## Conclusion

We demonstrate that establishment of ESCs requires upregulation of pluripotency-related genes and downregulation of differentiation-affiliated genes. Moreover, maintaining of DNA methylation at low levels established the ground-state pluripotency. In addition, we show the importance of the medium during the early days of ESC derivation which enables the capture of ESCs from blastocysts by maintaining the ground state of pluripotency. 

## Supplementary PDF


