# Supplementary material for: Temporal Gene Expression and DNA Methylation during Embryonic Stem Cell Derivation
Source: Cell J. 2018 May 28;20(3):361–8. doi: 10.22074/cellj.2018.5482 (PMC6004995; doi:10.22074/cellj.2018.5482)
Supplement: Supplementary file 1 [file Cell-J-20-361-s01.pdf]

CELL JOURNAL (Yakhteh)

Supplementary Information for

Temporal Gene Expression and DNA Methylation during Embryonic Stem Cell Derivation

Azam Samadian, M.Sc.[1#], Mahdi Hesaraki, M.Sc.[1#], Sepideh Mollamohammadi, M.Sc.[1], Behrouz Asgari, M.Sc.[1], Mehdi Totonchi, Ph.D.[1, 2*], Hossein Baharvand, Ph.D.[1, 3*]

1. Department of Stem Cells and Developmental Biology, Cell Science Research Center, Royan Institute for Stem Cell Biology and Technology, ACECR, Tehran, Iran
2. Department of Genetics, Reproductive Biomedicine Research Center, Royan Institute for Reproductive Biomedicine, ACECR, Tehran, Iran
3. Department of Developmental Biology, University of Science and Culture, Tehran, Iran

#The first two authors equally contributed to this article.

**Corresponding Address: P.O.Box: 16635-141, Department of Stem Cells and Developmental Biology, Cell Science Research Center, Royan Institute for Stem Cell Biology and Technology, ACECR, Tehran, Iran*
Emails: m.totonchi@royaninstitue.org, baharvand@royaninstitute.org

Table S1: The characteristics of primers used in quantitative real time-polymerase chain reaction analysis

Symbol	Gene sequences (5´-3´)	Annealing temperature (˚C)	Size (bp)	Accession number
Sox2	F: GCTGGGAGAAAGAAGAGGAG	63	180	NM_011443.3
	R: ATCTGGCGGAGAATAGTTGG			
Oct4	F: GCGTTCTCTTTGGAAAGGTG	62	204	NM_013633.2
	R: CGGTTCTCAATGCTAGTTCG			
Nanog	F: CTGATTCTTCTACCAGTCCCA	62	235	NM_028016.2
	R: AAACCAGGTCTTAACCTGCTTAT			
Rex1	F: TAGCCGCCTAGATTTCCACT	62	125	NM_009556.3
	R: GTCCATTTCTCTAATGCCCAC			
Utf1	F: ACCCTTCGATAACCAGATCC	62	219	NM_009482.2
	R: GGAAGAACTGAATCTGAGCG			
Sall4	F: CCACGAAAGGCAACCTGAAG	63	214	NM_175303.3
	R: TCTCTTTCCCTCAGCACTCA			
Tcf3	F: CAGCAGTGACCAGAACAGT	62	195	NM_001164147.1
	R: GAAGCCAGCCTGACTCAAG			
Dax1	F: CTCTTTAACCCAGACCTGCC	62	180	NM_007430.4
	R: CAGTGACGACATCGCTATTGA			
Dppa3	F: CTTTGTTGTCGGTGCTGAAA	62	100	NM_139218.1
	R: GTCCCGTTCAAACTCATTTCC			
β-Catenin	F: CATCTGTGCTCTTCGTCATC	61	145	NM_007614
	R: ATCCAACAGTTGCCTTTATCA			
Nodal	F: CATCCTACCAACCATGCCT	61	169	NM_013611.3
	R: CCACACTCCTCCACAATCA			
Gapdh	F: GACTTCAACAGCAACTCCCAC	60	125	NM_008084
	R: TCCACCACCCTGTTGCTGTA			
Cdx2	F: CTCCGAGAGGCAGGTTAA	61	109	NM_007673.3
	R: CTGTGGAGGCTGTTGTTG			
Gata 6	F: CACCACCATCACCATCAC	62	166	NM_010258.3
	R: AGGTCCTCCAACAGGTCT			
Lefty2	F: CAGCCAGAATTTTCGAGAGGT	61	248	NM_177099
	R: CAGTGCGATTGGAGCCATC			
Tet1	F: GACATCCCACAGACCGAAGA	62	146	NM_027384.1
	R: TTTCACTCCTCCCAAACTTACAG			
SetDB1	F: CTAATATGGGTGCTGTGAGGA	61	135	NM_001163642.1
	R: AGAGGAGGTATAGGAAGTGGG			
Carm1	F: TTTGCACAGGATAGAAATCCCAT	62	183	NM_021531.6
	R: AAACAACGGTGACTGGAAGAG			
